# Causal relationship between anti-inflammatory drugs and cancer: a pan-cancer study with Mendelian randomization

**DOI:** 10.3389/fgene.2024.1392745

**Published:** 2024-05-24

**Authors:** Shen Gao, Guojiang Wei, Qianwang Ma, Xue Wang, Sen Wang, Yuanjie Niu

**Affiliations:** ^1^ Tianjin Institute of Urology, The Second Hospital of Tianjin Medical University, Tianjin, China; ^2^ Department of Urology, The Second Hospital of Tianjin Medical University, Tianjin, China; ^3^ Department of Neurosurgery, First Affiliated Hospital of Harbin Medical University, Harbin, China

**Keywords:** Mendelian randomization analysis, NSAIDs, aspirin, anilide, pancancer, anti-inflammatory medications

## Abstract

**Background:**

Numerous epidemiological studies have elucidated the intricate connection between inflammation and cancer, highlighting how sustained inflammatory responses can fuel carcinogenesis by fostering proliferation, angiogenesis, and metastasis, while dampening immune responses and sensitivity to chemotherapy. Previous clinical investigations have underscored the potential of anti-inflammatory medications in either preventing or mitigating tumor formation. Here, the causal relationship between anti-inflammatory drugs and cancer was further explored through Mendelian randomization studies.

**Methods:**

Employing Mendelian randomization, we scrutinized the causal links between three anti-inflammatory drugs—NSAIDs, Aspirin, and Anilide—and 37 types of cancer. We primarily utilized inverse variance weighting (IVW) as the primary analytical approach to delineate the causal association between these drugs and cancer types. Concurrently, sensitivity analyses were conducted to ascertain the absence of horizontal pleiotropy and heterogeneity.

**Results:**

Our investigation revealed a discernible causal relationship between certain anti-inflammatory drugs and a subset of cancers, albeit without a pervasive impact across all cancer types. Specifically, NSAIDs exhibited a risk-reducing effect on non-small cell lung cancer (OR: 0.76, 95% CI: 0.59–0.97, *p*-value: 0.03) and gastric cancer (OR: 0.57, 95% CI: 0.34–0.98, *p*-value: 0.04). Conversely, aspirin was associated with an increased risk of oral malignant tumors (OR: 2.18, 95% CI: 1.13–4.21, *p*-value: 0.02). Notably, no statistically significant findings were observed for anilide drugs (*p* < 0.05).

**Conclusion:**

We identified several cancers with potential causal links to NSAIDs, including non-small cell lung cancer and gastric cancer. Despite our extensive analysis, we did not identify a substantial causal relationship between the use of anti-inflammatory drugs and the development of various cancers.

## 1 Introduction

Inflammation plays a pivotal role in cancer development, a concept first proposed by Rudolph Virchow in 1863 (Balkwill and Mantovani, 2001). Since then, the relationship between inflammation and carcinogenesis has gained widespread acceptance (Coussens and Werb, 2002; Perwez Hussain and Harris, 2007). Numerous studies have demonstrated a direct association between inflammation and the onset of various cancers (Wang et al., 2014; Hasche et al., 2017).

Given the strong correlation between inflammation and cancer, numerous studies have demonstrated the potential of common anti-inflammatory drugs, such as NSAIDs, in cancer prevention and risk reduction. Long-term NSAID use has been associated with decreased incidence of colorectal cancer, esophageal cancer, breast cancer, and lung cancer (Chan et al., 2005; Pereg and Lishner, 2005; Khandia and Munjal, 2020). Additionally, NSAIDs can enhance sensitivity to conventional therapies while reducing cancer invasion and metastasis (Zappavigna et al., 2020).

Indeed, various anti-inflammatory drugs exert their effects through different mechanisms. NSAIDs, for instance, primarily reduce inflammation by inhibiting cyclooxygenase (COX), which consequently impedes the conversion of arachidonic acid into thromboxane and prostaglandins (Vane, 1971; Bindu et al., 2020).

The efficacy of anti-inflammatory drugs in preventing and reducing cancer incidence remains an area of ongoing investigation, with varied conclusions across clinical studies. Inconsistent findings can often be attributed to inspection bias, unavoidable biases, and confounding factors, all of which contribute to the complexity of determining the relationship between anti-inflammatory drugs and cancer (Mahmud et al., 2004; Menter and Bresalier, 2023).

Navigating the relationship between anti-inflammatory drugs and cancer in real-world settings poses inherent challenges due to confounding factors and experimental design limitations. Mendelian randomization (MR) studies, employing genetic variation as instrumental variables (IVs), offer a promising avenue to mitigate biases arising from confounding factors and reverse causality (Katan, 2004; Lawlor et al., 2008). Thus, In this study we leveraged Mendelian randomization to investigate the causal relationship between anti-inflammatory drugs and cancer, with the aim of shedding new light on the potential role of anti-inflammatory drugs in cancer prevention and treatment.

## 2 Materials and methods

### 2.1 Data acquisition and aggregation

In this study, the anti-inflammatory drugs under investigation—NSAIDs, aniline drugs, and aspirin—were sourced from Wu et al. (2019)’s study, which aggregated GWAS data from the UK Biobank. This cohort comprised 502,616 participants, with approximately 54% being female and a mean age of 56.53 years (SD 8.09) (Wu et al., 2019). The mean body mass index (BMI) was 27.43 (SD 4.80). Study cohort characteristics were presented in Supplementary Table S4. Medications and diagnoses were obtained through nurse-led interviews. Only regular medications and supplements taken weekly, monthly, or three monthly were recorded. UK Biobank did not collect specific dosage and duration of the medications (Wu et al., 2019). Medications were classified using the Anatomical Therapeutic Chemical (ATC) classification system (Santos et al., 2017). Categories named by active ingredients were mapped directly to ATC codes. Categories named by brand name were first mapped to their active ingredients and then further mapped to ATC codes based on dosage and route of administration (if available). To ensure minimal sample overlap and facilitate cross-sectional comparability across studies, our outcome variables were derived from the Finnish project (Kurki et al., 2023). This encompassed data on 37 cancer types, including subtypes, and cancer summary GWAS data, accessible at https://www.finngen.fi/en.

### 2.2 Research design

In our Mendelian randomization study, we employed rigorous methods to explore the potential causal relationship between anti-inflammatory drugs and cancer. We adhered to three fundamental assumptions when selecting instrumental variables (Davey Smith and Hemani, 2014): 1. Utilizing SNPs strongly correlated with exposure factors as instrumental variables; 2. Ensuring instrumental variables are independent of potential confounding factors that could influence exposure and outcomes; 3. Verifying that the selected SNPs do not influence results through pathways other than the exposure under investigation.

**FIGURE 1:**
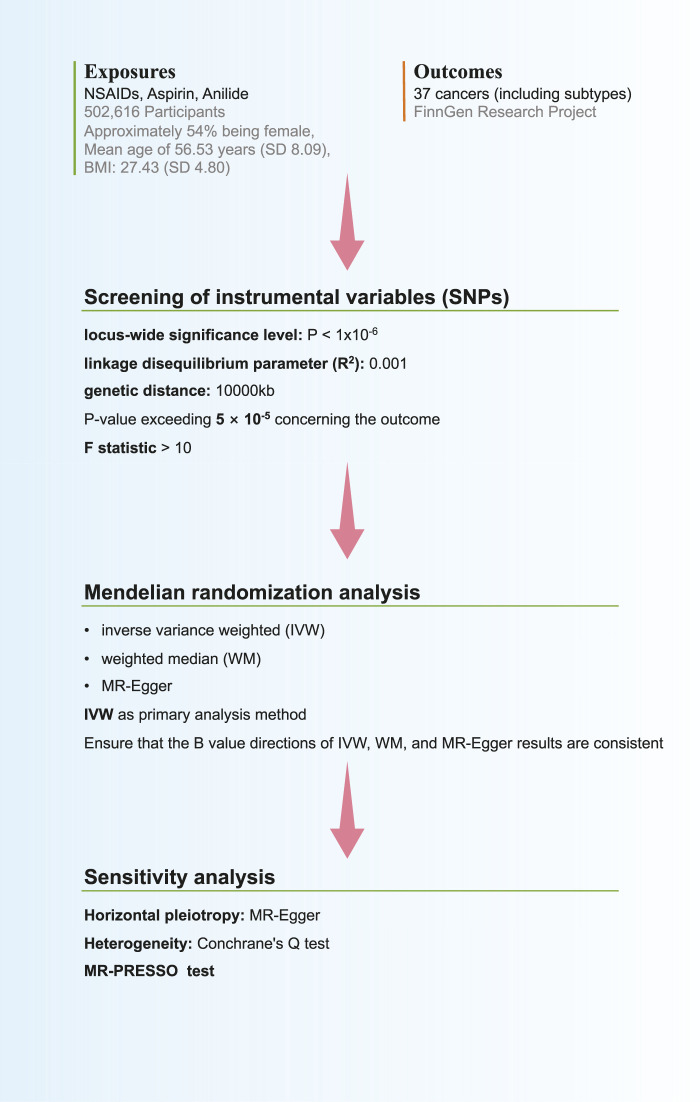
Flow chart of Mendelian randomization analysis in this study.

We applied three primary analysis methods: Inverse variance weighting (IVW), MR-Egger, and weighted median methods. Initially, we confirmed consistency in the direction of B values across IVW, MR-Egger, and weighted median results, prioritizing IVW results for the main analysis (Bowden et al., 2017). Furthermore, sensitivity analysis was conducted to concurrently assess the presence of horizontal pleiotropy and heterogeneity. A *p*-value exceeding 0.05 for MR-Egger indicated the absence of significant horizontal pleiotropy. Heterogeneity was evaluated using Conchrane’s Q test, with a *p*-value exceeding 0.05 suggesting the absence of significant heterogeneity. Additionally, leave-one-out sensitivity analyses were conducted to evaluate result robustness. The MR pleiotropy residual sum and outliers (MR-PRESSO) test was used to further confirm the existence of horizontal pleiotropy, and MR-PRESSO has higher accuracy and can be used to identify horizontal pleiotropy and outliers (*p* > 0.05 indicates no horizontal pleiotropy) (Figure 1).

All analyses were performed using the TwoSampleMR R package, with statistical significance set at *p* < 0.05.

### 2.3 Instrument variable selection

To ensure robustness in our selection of instrumental variables, we adopted stringent criteria. We selected single nucleotide polymorphisms (SNPs) with a *p*-value below the locus-wide significance threshold of 1 × 10^−6^ as instrumental variables. Additionally, we set the SNP linkage disequilibrium parameter (*R* ^2^) to 0.001 to ensure independence among selected SNPs. To further guarantee the independence of instrumental variables, we maintained a genetic distance of 10,000 kb between them. Moreover, we verified that the *p*-values of both instrumental variables and outcomes exceeded 5 × 10^−5^, ensuring that only SNPs strongly associated with exposure factors and outcomes were included in our analysis. To guarantee the strength of the selected IVs, we used the variance (*R* ^2^) and F statistics. *R* ^2^ was calculated as follows: 2× (1−MAF) ×MAF × β^2^ (MAF, minor allele frequency; β, effect size of exposure). The F statistic is calculated using formula F = *R* ^2^(N−2)/(1−*R* ^2^). Among them, *R* ^2^ represents the proportion of exposure variance explained by the independent variable, and N represents the sample size. An F statistic >10 indicates a strong correlation between IV and exposure. The instrumental variables (SNPs) related to exposures included in this study were in Supplementary Table S5.

## 3 Result

### 3.1 Causal relationship between NSAIDs and cancer

**FIGURE 2:**
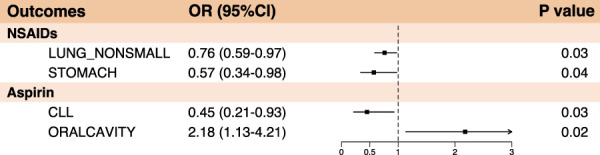
Forest plot of statistically significant summary results in Mendelian randomization analysis of anti-inflammatory drugs and cancer (*p* < 0.05).

**FIGURE 3:**
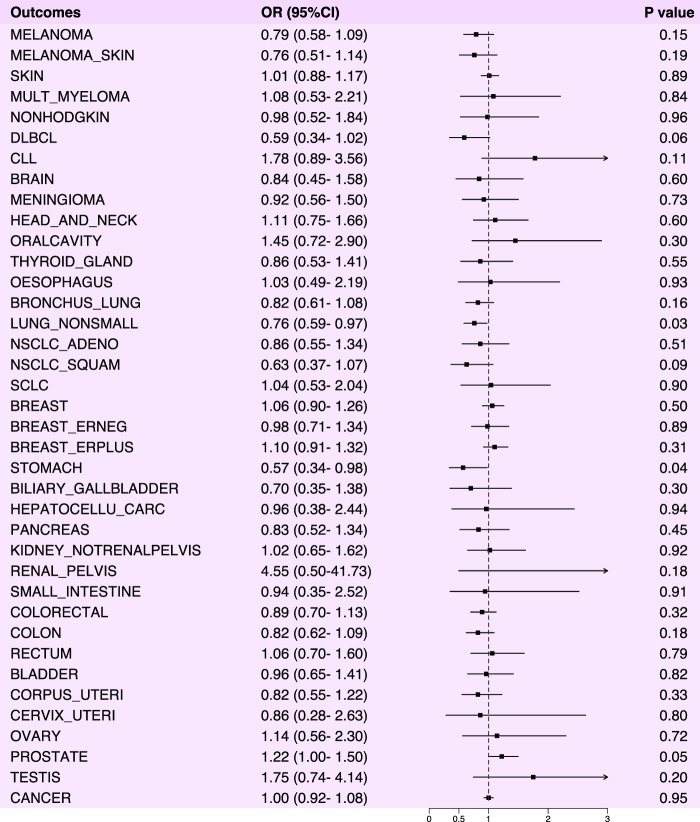
Forest plot of Mendelian randomization analysis results between NSAIDs and various cancers.

In our analysis of the causal relationship between NSAIDs and various cancers, we observed statistically significant risk reductions for non-small cell lung cancer (OR: 0.76, 95% CI: 0.59–0.97, *p*-value: 0.03) and gastric cancer (OR: 0.57, 95% CI: 0.34–0.98, *p*-value: 0.04) (Figure 2). We also performed horizontal pleiotropy and heterogeneity analyzes to assess potential bias in their results. The analysis showed that the selected SNPs did not exhibit horizontal pleiotropy or heterogeneity, as evidenced by a *p*-value greater than 0.05 (Supplementary Table S1). Overall, NSAIDs exhibited a risk-reducing effect (OR<1) in 22 cancers and cancer pools, including ER-breast (OR: 0.98, 95% CI: 0.71–1.34, *p*-value: 0.89), colorectal cancer (OR: 0.89, 95% CI: 0.70–1.13, *p*-value: 0.32), colon cancer (OR: 0.82, 95% CI: 0.62–1.09, *p*-value: 0.18), and cervical cancer (OR: 0.86, 95% CI: 0.28–2.63, *p*-value: 0.80), among others. However, NSAIDs demonstrated an increased risk effect for prostate cancer (OR: 1.22, 95% CI: 1.00–1.50, *p*-value: 0.05) (Figure 3; Supplementary Table S1).

### 3.2 Causal link between aspirin and cancer

**FIGURE 4:**
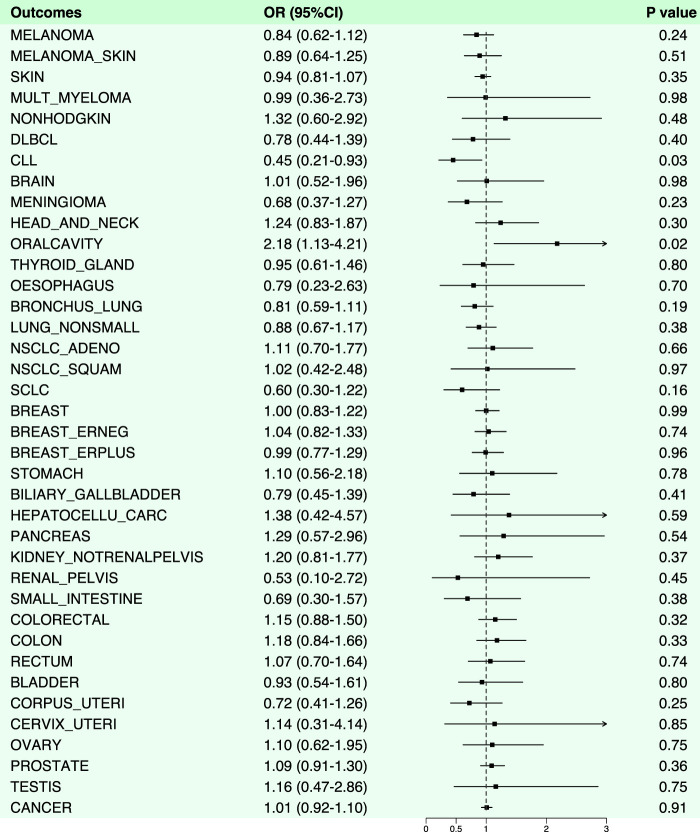
Forest plot of results from Mendelian randomization analysis between aspirin and various cancers.

Aspirin was demonstrated a protective effect against chronic lymphocytic leukemia (OR: 0.45, 95% CI: 0.21–0.93, *p*-value: 0.03). However, it exhibited an increased risk effect against oral malignancies (OR: 2.18, 95% CI: 1.13–4.21, *p*-value: 0.02) (Figures 2, 4; Supplementary Table S2). Notably, in the analysis results with chronic lymphocytic leukemia, the B value of MR-Egger was >0, suggesting that the result lacked robustness. Likewise, we also performed horizontal pleiotropy and heterogeneity analyzes to assess potential biases in this part. The analysis showed that the selected SNPs did not exhibit horizontal pleiotropy or heterogeneity, as evidenced by a *p*-value greater than 0.05.

### 3.3 Causal relationship between anilide drugs and cancer

**FIGURE 5:**
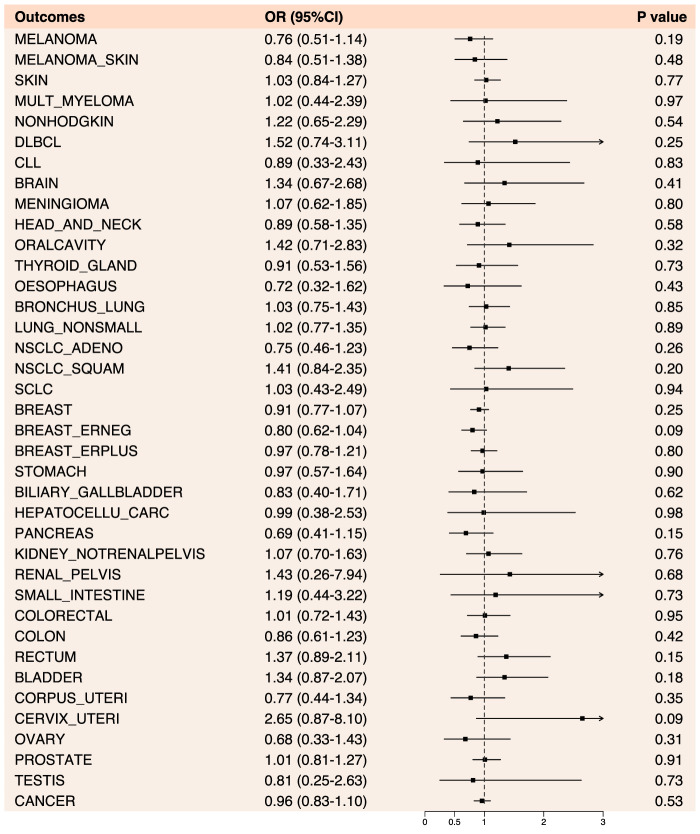
Forest plot of results from Mendelian randomization analysis between anilide drugs and various cancers.

In the Mendelian randomization analysis of anilide and cancer, no statistically significant results were observed. Overall, anilide drugs exhibited a risk-reducing effect (OR < 1) for 18 cancers and cancer pools, including breast (OR: 0.91, 95% CI: 0.77–1.07, *p*-value: 0.25), colon cancer (OR: 0.86, 95% CI: 0.61–1.23, *p*-value: 0.42), among others. Conversely, they showed an increased risk effect (OR > 1) for 19 types of cancer, including prostate cancer (OR: 1.01, 95% CI: 0.81–1.27, *p*-value: 0.91) (Figure 5; Supplementary Table S3).

## 4 Discussion

The connection between chronic inflammation and cancer is widely acknowledged. Numerous epidemiological studies have provided compelling evidence of a robust correlation between inflammation and cancer (Hoepelman, 2001). This association stems from the ability of inflammation to foster a microenvironment conducive to cancer cell proliferation, migration, and metastasis, thus facilitating the development of cancer (Khandia and Munjal, 2020). Chronic inflammation, characterized by heightened cell proliferation and impaired DNA repair mechanisms, is particularly implicated in elevating cancer risk (Guo et al., 2017).

The profound connection between inflammation and cancer underscores the rationale for utilizing anti-inflammatory drugs as a therapeutic strategy. Aspirin and NSAIDs, long established in clinical practice, offer multifaceted benefits beyond their anti-inflammatory, antipyretic, and analgesic properties, including cardioprotective effects. Emerging evidence further suggests their potential in cancer prevention and risk reduction (Cuzick et al., 2009; Kolawole and Kashfi, 2022).

Indeed, NSAIDs exert their anti-inflammatory effects primarily by inhibiting cyclooxygenase, which plays a pivotal role in the synthesis of prostaglandins and thromboxane from arachidonic acid (Simmons et al., 2004; Panchal and Prince Sabina, 2023). However, their impact extends beyond mere inflammation, as NSAIDs have been implicated in anticancer activities through diverse mechanisms (Kopp and Ghosh, 1994; Arico et al., 2002; Shureiqi et al., 2003; Gurpinar et al., 2014; Kolawole and Kashfi, 2022).

Our study delves into the potential causal association between anti-inflammatory drugs and cancer through Mendelian randomization analysis, aiming to illuminate novel insights into their interplay. In our examination of NSAIDs and cancer, we observed protective effects against non-small cell lung cancer (OR: 0.76, 95% CI: 0.59–0.97, *p*-value: 0.03) and gastric cancer (OR: 0.57, 95% CI: 0.34–0.98, *p*-value: 0.04), suggesting a potential avenue for their therapeutic utility (*p* < 0.05). Notably, COX-2, typically minimally expressed in normal lung tissue, was found to be overexpressed in specimens from non-small cell lung cancer (NSCLC) patients, correlating with poorer prognosis (Khuri et al., 2001; Petkova et al., 2004; Sandler and Dubinett, 2004). Clinical evidence further supported the notion that NSAIDs may exert protective effects against lung cancer through pathways beyond mere anti-inflammatory actions. Although research on NSAIDs and gastric cancer remains limited, a meta-analysis has demonstrated aspirin’s efficacy in mitigating the risk of non-cardia gastric cancer and *Helicobacter pylori* infection (Yang et al., 2010). Elevated COX-2 expression in gastric cancer underscores its potential etiological role, with studies indicating a reduction in COX-2 expression following *H. pylori* eradication (Ji et al., 2021).

In our Mendelian randomization study concerning aspirin and cancer, we uncovered a risk-increasing effect on oral malignancies (OR: 2.18, 95% CI: 1.13–4.21, *p*-value: 0.02), a finding that currently lacks robust research support. Regarding the extensively studied impact of NSAIDs on colorectal cancer prevention, our analysis revealed no statistically significant protective effect for either NSAIDs (OR: 0.89, 95% CI: 0.70–1.13, *p*-value: 0.32) or aspirin alone (OR: 1.15, 95% CI: 0.88–1.50, *p*-value: 0.32). The onset of colorectal cancer is intricately linked to various factors, encompassing COX activity, platelet-mediated effects, inflammation, intestinal microorganisms, and more. The multifaceted effects of NSAIDs hint at complex underlying mechanisms yet to be fully elucidated. Similarly, the nuanced biochemistry of aspirin may yield divergent outcomes. Notably, the US Preventive Services Task Force’s recent revisions suggested insufficient evidence to support the notion that low-dose aspirin reduces mortality from colorectal cancer (Dehmer et al., 2022; US Preventive Services Task Force et al., 2022).

While in our Mendelian randomization analysis examining the association between aniline anti-inflammatory drugs and cancer, we did not observe any statistically significant results. Aniline anti-inflammatory drugs, characterized as relatively mild analgesics and antipyretics, are exemplified by acetaminophen, which has been a mainstay in clinical use for over a century. Despite its long history, the exact mechanism of action remains elusive. Traditionally, it was believed to share a mechanism akin to classic non-steroidal anti-inflammatory drugs (NSAIDs) by inhibiting COX enzymes (Graham et al., 2013). However, emerging evidence suggested distinctions from traditional NSAIDs as it largely lacks peripheral anti-inflammatory effects. Notably, when levels of arachidonic acid and peroxide are elevated, its activity is minimal, resulting in weak anti-inflammatory effects (Ghanem et al., 2016). Consequently, the limited breadth of COX enzyme inhibitory activity may undermine the efficacy of aniline anti-inflammatory drugs against inflammation and cancer.

The pharmacological differences among NSAIDs, Aspirin, and Anilide may be closely associated with their anticancer effects. Acetaminophen, the most common Anilide, is widely utilized for its antipyretic and analgesic properties. Its pharmacokinetic characteristics support its centrally mediated mechanism of action (Ayoub, 2021). Acetaminophen is essentially nonionizable within the physiological pH range, and its lipid solubility facilitates rapid penetration of cell membranes and easy passage across the blood-brain barrier (Clissold, 1986). Consequently, acetaminophen primarily exerts its antipyretic effect by inhibiting the synthesis and release of prostaglandins in the hypothalamus, resulting in weaker peripheral effects (Seppälä et al., 1983; Muth-Selbach et al., 1999).

Studies have demonstrated that anti-inflammatory drugs, such as NSAIDs, can modulate the pharmacokinetics of other medications (Zappavigna et al., 2020). By altering the metabolism of these drugs, they can enhance efficacy and reduce toxicity. For instance, diclofenac inhibits the glucuronidation of DMXAA, thereby impeding its metabolism and elevating its plasma concentration (Wang et al., 2009).

In addition to their ability to reduce the toxicity of conventional drugs by altering their metabolism, the effective anticancer effects of NSAIDs when combined with chemotherapy drugs may also be attributed to their chemosensitizing effects (Thiruchenthooran et al., 2023). Several preclinical studies have demonstrated that the combination of celecoxib with etoposide, doxorubicin, vincristine, or irinotecan can result in additive or synergistic effects *in vitro* (Ponthan et al., 2007; Arunasree et al., 2008). Moreover, celecoxib enhances the response of prostate cancer cells to docetaxel both *in vitro* and *in vivo* (Nakata et al., 2004). Currently, combining chemotherapy drugs with NSAIDs provides a promising approach to enhancing drug activity and increasing the lipophilicity of chemotherapy agents (Ravera et al., 2019).

In our study, we delved into the potential causal relationship between NSAIDs anti-inflammatory drugs and cancer using Mendelian randomization analysis. Our findings unveiled a risk-reducing effect of NSAIDs on non-small cell lung cancer and gastric cancer, while conversely, aspirin exhibited an increased risk effect on oral cancer. Notably, anilide did not yield statistically significant results. The comprehensive analysis did not reveal a widespread risk-reducing impact of anti-inflammatory drugs on cancer. The application of Mendelian randomization analysis enabled effective control over the influence of confounding factors. Given the multifaceted mechanisms of action of NSAIDs drugs, our research contributes valuable insights to existing clinical studies and charts new directions for future research endeavors.

While our study provides valuable insights, it’s important to acknowledge its limitations. Firstly, the data primarily focused on European populations, potentially limiting the generalizability of our findings to other ethnic groups. Additionally, human factors during data acquisition, processing, and analysis may have influenced the accuracy of our results. These limitations highlight the need for further research involving diverse populations and meticulous data handling to ensure robust and comprehensive findings.

## 5 Conclusion

In our Mendelian randomization study, we delved into the causal association between NSAIDs anti-inflammatory drugs and cancer. Despite our extensive analysis, we found that only a select few cancers exhibited a risk-reducing effect, leading us to refrain from concluding that NSAIDs anti-inflammatory drugs possess a broad protective effect against cancer.

## Figures and Tables

**Table:** 

**MELANOMA**	Malignant melanoma
**MELANOMA_SKIN**	Malignant melanoma of skin
**SKIN**	Malignant neoplasm of skin
**MULT_MYELOMA**	Multiple myeloma
**NONHODGKIN**	Non-Hodgkin lymphoma, all
**DLBCL**	Diffuse large B-cell lymphoma
**CLL**	Chronic lymphocytic leukaemia
**BRAIN**	Malignant neoplasm of brain
**MENINGIOMA**	Brain meningioma
**HEAD_AND_NECK**	Malignant neoplasm of head and neck
**ORALCAVITY**	Malignant neoplasm of oral cavity
**THYROID_GLAND**	Malignant neoplasm of thyroid gland
**OESOPHAGUS**	Malignant neoplasm of oesophagus
**BRONCHUS_LUNG**	Malignant neoplasm of bronchus and lung
**LUNG_NONSMALL**	Non-small cell lung cancer
**NSCLC_ADENO**	Non-small cell lung cancer, adenocarcinoma
**NSCLC_SQUAM**	Non-small cell lung cancer, squamous
**SCLC**	Small cell lung cancer
**BREAST**	Malignant neoplasm of breast
**BREAST_ERNEG**	Malignant neoplasm of breast, HER-negative
**BREAST_ERPLUS**	Malignant neoplasm of breast, HER-positive
**STOMACH**	Malignant neoplasm of stomach
**BILIARY_GALLBLADDER**	Malignant neoplasm of intrahepatic ducts, biliary tract and gallbladder
**HEPATOCELLU_CARC**	Hepatocellular carcinoma
**PANCREAS**	Malignant neoplasm of pancreas
**KIDNEY_NOTRENALPELVIS**	Malignant neoplasm of kidney, except renal pelvis
**RENAL_PELVIS**	Malignant neoplasm of renal pelvis
**SMALL_INTESTINE**	Malignant neoplasm of small intestine
**COLORECTAL**	Colorectal cancer
**COLON**	Malignant neoplasm of colon
**RECTUM**	Malignant neoplasm of rectum
**BLADDER**	Malignant neoplasm of bladder
**CORPUS_UTERI**	Malignant neoplasm of corpus uteri
**CERVIX_UTERI**	Malignant neoplasm of cervix uteri
**OVARY**	Malignant neoplasm of ovary
**PROSTATE**	Malignant neoplasm of prostate
**TESTIS**	Malignant neoplasm of testis
**CANCER**	Malignant neoplasm

## Data Availability

The original contributions presented in the study are included in the article/Supplementary Material, further inquiries can be directed to the corresponding author.
