# Supplementary material for: Causal relationship between anti-inflammatory drugs and cancer: a pan-cancer study with Mendelian randomization
Source: Front Genet. 2024 May 24;15:1392745. doi: 10.3389/fgene.2024.1392745 (PMC11156997; doi:10.3389/fgene.2024.1392745)
Supplement: Supplementary file 1 [file DataSheet1.zip › Supplementary Materials/Supplementary Table S1-S3.docx]

|  | **Conchrane's Q test** | | **MR-Egger** | | **MR-PRESSO** | |
| --- | --- | --- | --- | --- | --- | --- |
| **Outcomes** | **Q value** | **P value** | **Egger intercept** | **P value** | **RSS** | **P value** |
| MELANOMA | 21.61 | 0.20 | -0.0263 | 0.40 | 24.70 | 0.23 |
| MELANOMA_SKIN | 26.91 | 0.06 | -0.0382 | 0.34 | 31.23 | 0.07 |
| SKIN | 21.45 | 0.21 | -0.0027 | 0.85 | 23.98 | 0.25 |
| MULT_MYELOMA | 14.09 | 0.66 | -0.0825 | 0.25 | 17.24 | 0.63 |
| NONHODGKIN | 21.67 | 0.20 | 0.0272 | 0.67 | 24.33 | 0.23 |
| DLBCL | 15.79 | 0.54 | 0.0514 | 0.34 | 18.22 | 0.58 |
| CLL | 8.29 | 0.96 | 0.0820 | 0.24 | 10.91 | 0.92 |
| BRAIN | 15.25 | 0.58 | 0.0178 | 0.77 | 17.24 | 0.65 |
| MENINGIOMA | 17.47 | 0.42 | 0.0333 | 0.50 | 20.09 | 0.44 |
| HEAD_AND_NECK | 18.45 | 0.36 | 0.0480 | 0.22 | 22.32 | 0.36 |
| ORALCAVITY | 21.61 | 0.20 | 0.0544 | 0.43 | 25.20 | 0.21 |
| THYROID_GLAND | 25.27 | 0.09 | 0.0022 | 0.96 | 27.63 | 0.14 |
| OESOPHAGUS | 18.24 | 0.37 | 0.0634 | 0.40 | 21.36 | 0.41 |
| BRONCHUS_LUNG | 26.42 | 0.07 | 0.0053 | 0.85 | 29.48 | 0.10 |
| LUNG_NONSMALL | 15.83 | 0.54 | 0.0086 | 0.73 | 17.70 | 0.61 |
| NSCLC_ADENO | 12.46 | 0.77 | -0.0146 | 0.74 | 14.04 | 0.81 |
| NSCLC_SQUAM | 23.56 | 0.13 | 0.0270 | 0.62 | 26.40 | 0.17 |
| SCLC | 10.99 | 0.86 | 0.0199 | 0.76 | 12.49 | 0.88 |
| BREAST | 22.60 | 0.16 | -0.0064 | 0.71 | 25.57 | 0.19 |
| BREAST_ERNEG | 33.02 | 0.01 | -0.0043 | 0.89 | 37.19 | 0.02 |
| BREAST_ERPLUS | 7.98 | 0.97 | -0.0102 | 0.57 | 9.37 | 0.96 |
| STOMACH | 21.76 | 0.19 | 0.0024 | 0.96 | 24.11 | 0.26 |
| BILIARY_GALLBLADDER | 30.37 | 0.02 | 0.0102 | 0.88 | 34.68 | 0.03 |
| HEPATOCELLU_CARC | 22.14 | 0.18 | -0.0793 | 0.39 | 25.58 | 0.21 |
| PANCREAS | 16.06 | 0.52 | 0.0793 | 0.09 | 21.95 | 0.35 |
| KIDNEY_NOTRENALPELVIS | 27.53 | 0.05 | -0.0067 | 0.88 | 29.85 | 0.09 |
| RENAL_PELVIS | 37.11 | 0.00 | 0.1262 | 0.57 | 41.65 | 0.01 |
| SMALL_INTESTINE | 28.88 | 0.04 | 0.0022 | 0.98 | 31.76 | 0.06 |
| COLORECTAL | 11.02 | 0.86 | 0.0675 | 0.01 | 24.18 | 0.27 |
| COLON | 12.25 | 0.78 | 0.0351 | 0.21 | 16.01 | 0.71 |
| RECTUM | 11.03 | 0.86 | 0.1222 | 0.00 | 26.78 | 0.17 |
| BLADDER | 18.00 | 0.39 | 0.0128 | 0.75 | 19.97 | 0.47 |
| CORPUS_UTERI | 11.36 | 0.84 | 0.0178 | 0.65 | 12.68 | 0.90 |
| CERVIX_UTERI | 27.34 | 0.05 | 0.0311 | 0.78 | 30.28 | 0.09 |
| OVARY | 30.02 | 0.03 | 0.0237 | 0.74 | 34.19 | 0.03 |
| PROSTATE | 22.91 | 0.15 | 0.0229 | 0.25 | 27.22 | 0.17 |
| TESTIS | 14.88 | 0.60 | 0.0225 | 0.79 | 17.28 | 0.64 |
| CANCER | 16.51 | 0.49 | 0.0108 | 0.16 | 21.06 | 0.40 |

Table 1. Results of Mendelian Randomization Sensitivity Analysis between NSAIDs and Cancer.

Table 2. Results of Mendelian Randomization Sensitivity Analysis between Aspirin and Cancer.

|  | **Conchrane's Q test** | | **MR-Egger** | | **MR-PRESSO** | |
| --- | --- | --- | --- | --- | --- | --- |
| **Outcomes** | **Q value** | **P value** | **Egger intercept** | **P value** | **RSS** | **P value** |
| MELANOMA | 5.76 | 0.67 | 0.0263 | 0.39 | 7.82 | 0.72 |
| MELANOMA_SKIN | 6.07 | 0.64 | 0.0014 | 0.97 | 7.72 | 0.75 |
| SKIN | 6.28 | 0.62 | 0.0040 | 0.78 | 8.22 | 0.68 |
| MULT_MYELOMA | 16.43 | 0.04 | -0.0151 | 0.89 | 20.74 | 0.05 |
| NONHODGKIN | 11.22 | 0.19 | -0.1203 | 0.12 | 18.30 | 0.09 |
| DLBCL | 6.65 | 0.58 | -0.0066 | 0.91 | 8.16 | 0.66 |
| CLL | 2.54 | 0.96 | -0.1060 | 0.18 | 5.70 | 0.89 |
| BRAIN | 6.61 | 0.58 | -0.0615 | 0.37 | 9.69 | 0.55 |
| MENINGIOMA | 10.85 | 0.21 | -0.0656 | 0.30 | 16.07 | 0.20 |
| HEAD_AND_NECK | 9.35 | 0.31 | -0.0111 | 0.80 | 11.63 | 0.41 |
| ORALCAVITY | 5.51 | 0.70 | 0.0266 | 0.69 | 6.92 | 0.80 |
| THYROID_GLAND | 4.66 | 0.79 | 0.0583 | 0.21 | 8.34 | 0.67 |
| OESOPHAGUS | 21.82 | 0.01 | 0.0272 | 0.83 | 27.82 | 0.01 |
| BRONCHUS_LUNG | 12.45 | 0.13 | 0.0383 | 0.25 | 17.49 | 0.14 |
| LUNG_NONSMALL | 7.50 | 0.48 | 0.0394 | 0.17 | 11.77 | 0.40 |
| NSCLC_ADENO | 4.05 | 0.85 | 0.0307 | 0.52 | 5.50 | 0.88 |
| NSCLC_SQUAM | 28.90 | 0.00 | 0.0331 | 0.73 | 35.68 | 0.00 |
| SCLC | 7.65 | 0.47 | 0.0671 | 0.36 | 10.17 | 0.51 |
| BREAST | 12.80 | 0.12 | 0.0118 | 0.56 | 17.09 | 0.13 |
| BREAST_ERNEG | 8.08 | 0.43 | 0.0199 | 0.43 | 10.45 | 0.49 |
| BREAST_ERPLUS | 15.41 | 0.05 | 0.0088 | 0.75 | 19.68 | 0.07 |
| STOMACH | 13.45 | 0.10 | -0.0830 | 0.23 | 20.88 | 0.06 |
| BILIARY_GALLBLADDER | 5.89 | 0.66 | 0.0999 | 0.10 | 11.70 | 0.40 |
| HEPATOCELLU_CARC | 15.95 | 0.04 | 0.1111 | 0.37 | 23.29 | 0.03 |
| PANCREAS | 26.03 | 0.00 | 0.0094 | 0.92 | 35.13 | <0.001 |
| KIDNEY_NOTRENALPELVIS | 4.01 | 0.86 | -0.0313 | 0.43 | 5.81 | 0.87 |
| RENAL_PELVIS | 2.41 | 0.97 | -0.0688 | 0.68 | 3.20 | 0.98 |
| SMALL_INTESTINE | 4.38 | 0.82 | -0.1284 | 0.15 | 8.73 | 0.63 |
| COLORECTAL | 8.86 | 0.35 | 0.0404 | 0.13 | 13.96 | 0.28 |
| COLON | 9.88 | 0.27 | 0.0387 | 0.26 | 13.88 | 0.27 |
| RECTUM | 10.08 | 0.26 | 0.0390 | 0.37 | 13.34 | 0.30 |
| BLADDER | 13.90 | 0.08 | -0.0538 | 0.34 | 19.06 | 0.10 |
| CORPUS_UTERI | 13.19 | 0.11 | 0.0740 | 0.20 | 21.08 | 0.06 |
| CERVIX_UTERI | 16.32 | 0.04 | 0.0469 | 0.73 | 20.48 | 0.08 |
| OVARY | 8.15 | 0.42 | 0.0054 | 0.93 | 11.03 | 0.49 |
| PROSTATE | 7.85 | 0.45 | 0.0192 | 0.31 | 10.93 | 0.46 |
| TESTIS | 2.60 | 0.96 | -0.0052 | 0.95 | 3.19 | 0.98 |
| CANCER | 9.79 | 0.28 | 0.0069 | 0.45 | 12.99 | 0.35 |

Table 3. Results of Mendelian Randomization Sensitivity Analysis between Anilide and Cancer.

|  | **Conchrane's Q test** | | **MR-Egger** | | **MR-PRESSO** | |
| --- | --- | --- | --- | --- | --- | --- |
| **Outcomes** | **Q value** | **P value** | **Egger intercept** | **P value** | **RSS** | **P value** |
| MELANOMA | 25.93 | 0.03 | -0.0140 | 0.74 | 29.78 | 0.03 |
| MELANOMA_SKIN | 29.92 | 0.01 | -0.0002 | 1.00 | 33.10 | 0.02 |
| SKIN | 23.13 | 0.04 | 0.0186 | 0.37 | 28.33 | 0.05 |
| MULT_MYELOMA | 16.13 | 0.31 | 0.0807 | 0.35 | 19.47 | 0.33 |
| NONHODGKIN | 10.34 | 0.74 | 0.0964 | 0.14 | 14.64 | 0.64 |
| DLBCL | 16.62 | 0.22 | -0.0333 | 0.65 | 19.25 | 0.29 |
| CLL | 22.09 | 0.08 | -0.1418 | 0.15 | 28.96 | 0.04 |
| BRAIN | 8.19 | 0.88 | 0.0658 | 0.35 | 10.11 | 0.89 |
| MENINGIOMA | 11.45 | 0.65 | 0.0572 | 0.30 | 14.23 | 0.66 |
| HEAD_AND_NECK | 8.83 | 0.84 | 0.0244 | 0.56 | 10.70 | 0.87 |
| ORALCAVITY | 14.74 | 0.40 | -0.0003 | 1.00 | 17.39 | 0.44 |
| THYROID_GLAND | 19.14 | 0.16 | -0.0624 | 0.25 | 23.93 | 0.14 |
| OESOPHAGUS | 13.00 | 0.53 | 0.1125 | 0.17 | 16.86 | 0.48 |
| BRONCHUS_LUNG | 21.41 | 0.09 | -0.0369 | 0.26 | 26.71 | 0.07 |
| LUNG_NONSMALL | 10.55 | 0.72 | 0.0062 | 0.82 | 11.88 | 0.79 |
| NSCLC_ADENO | 9.12 | 0.82 | -0.0244 | 0.61 | 10.55 | 0.87 |
| NSCLC_SQUAM | 8.97 | 0.83 | 0.0068 | 0.89 | 10.10 | 0.90 |
| SCLC | 18.20 | 0.20 | -0.1233 | 0.15 | 24.12 | 0.16 |
| BREAST | 12.29 | 0.58 | 0.0137 | 0.41 | 14.72 | 0.60 |
| BREAST_ERNEG | 11.84 | 0.62 | 0.0008 | 0.97 | 13.59 | 0.70 |
| BREAST_ERPLUS | 16.43 | 0.29 | 0.0195 | 0.38 | 19.40 | 0.30 |
| STOMACH | 11.40 | 0.65 | 0.0305 | 0.57 | 13.49 | 0.67 |
| BILIARY_GALLBLADDER | 19.83 | 0.14 | 0.0989 | 0.17 | 25.71 | 0.10 |
| HEPATOCELLU_CARC | 15.34 | 0.36 | 0.0859 | 0.37 | 18.21 | 0.40 |
| PANCREAS | 11.80 | 0.62 | 0.0482 | 0.35 | 14.45 | 0.63 |
| KIDNEY_NOTRENALPELVIS | 15.98 | 0.31 | -0.0062 | 0.89 | 18.86 | 0.35 |
| RENAL_PELVIS | 13.20 | 0.51 | 0.1728 | 0.32 | 16.15 | 0.52 |
| SMALL_INTESTINE | 19.78 | 0.14 | -0.0460 | 0.65 | 23.17 | 0.18 |
| COLORECTAL | 27.38 | 0.02 | 0.0407 | 0.24 | 34.53 | 0.01 |
| COLON | 16.85 | 0.26 | 0.0458 | 0.19 | 21.74 | 0.22 |
| RECTUM | 16.71 | 0.27 | 0.0342 | 0.43 | 19.80 | 0.32 |
| BLADDER | 12.18 | 0.59 | -0.0016 | 0.97 | 13.67 | 0.68 |
| CORPUS_UTERI | 21.25 | 0.10 | -0.0746 | 0.18 | 27.14 | 0.06 |
| CERVIX_UTERI | 18.65 | 0.18 | -0.0311 | 0.79 | 21.21 | 0.27 |
| OVARY | 19.02 | 0.16 | 0.1160 | 0.11 | 26.78 | 0.09 |
| PROSTATE | 20.73 | 0.11 | -0.0157 | 0.50 | 24.02 | 0.11 |
| TESTIS | 22.88 | 0.06 | 0.0114 | 0.92 | 27.01 | 0.08 |
| CANCER | 41.93 | <0.001 | 0.0085 | 0.56 | 48.97 | <0.001 |
